# Risk Management in E-Commerce—A Fraud Study Case Using Acoustic Analysis through Its Complexity

**DOI:** 10.3390/e21111087

**Published:** 2019-11-07

**Authors:** Diego C. Nascimento, Bruno Barbosa, André M. Perez, Daniel O. Caires, Edgar Hirama, Pedro L. Ramos, Francisco Louzada

**Affiliations:** Institute of Mathematical Science and Computing, University of São Paulo, 13566-590 São Carlos, Brazil; brunobarbosa@usp.br (B.B.); andre.marcos.perez@usp.br (A.M.P.); dielcaires@usp.br (D.O.C.); edgar.hirama@usp.br (E.H.); louzada@icmc.usp.br (F.L.)

**Keywords:** sound analysis, acoustic indexes, entropy, fraud detection

## Abstract

This work aimed to develop business intelligence towards fraud detection using buyer-placed information combined with the sound analysis from a confirmation purchase call. We used a dataset of 789 orders in 2018, provided by different e-commerce websites and calls fulfilled from every Brazilian state. Nine acoustic index features were used, through entropy in sound and vibration, summarizing the audio plus 6 extra features related, added by 12 customer features to compose two different classifiers (Logistic Regression and Random Forest). The acoustic indexes were, in fact, capable of providing better accuracy of the models, showing a probability associated with the voice characteristics, helping decision-making in credit card fraud.

## 1. Introduction

According to Bhatla et al. [1], credit card fraud happens when some individual uses another individual’s credit card for his or her own benefit, whilst neither the card owner or the card issuer are aware of the fact that the card is being used. Furthermore, the fraudster has no connection with any of the other parties involved and no intention of either contacting the card owner or making repayments for the purchases made.

While credit card fraud has been increasing worldwide, there is a significant effort to try to reduce the losses for credit card companies and merchants. Detecting it poses some challenges, including the high number of transactions, the amount of data involved in each of them and the need to reduce the occurrences of false positives, which can overload the fraud department responsible for checking it, for example, via phone call to the cardholder [2]. One way is to focus on the records regarding the phone calls and other features, to classify possible fraudulent acts.

In this paper, we introduce an approach to sound analysis events in the fraud context, so that acoustic indices can be computed to improve the performance of the classifiers. Complemented by variables from the purchase data to try to find a correlation between them and the fraudsters’ behavior. Moreover, in this work we addressed the feature extraction task, related with the fraud detection using buyer-placed information combined with the sound analysis (and its complexity), as a business intelligence tool.

An audio can be interpreted as a wave signal in which the observations are indexed in time. Using the Fourier transform, it is possible to convert and segment to an equivalent representation in frequency domain. Therefore, this type of analysis represents a sum of sinusoidal waves, as a composition of simple harmonic vibrations, with specific frequencies. The frequency content is rich in intelligible information that can be used to differentiate between the signal sources by working out the differences between them.

Complementary to that, following Sueur et al. [3], the importance of sound as a raw material extends to many areas of science. Thus, the audio analysis can be summarized through indices, which represent its complexity, as well as phenomenon both in the domain of time and frequency.

This paper is organized as follows. In Section 2, we describe the data structure and adopted audio processing methodology and establish the basis for a theoretical approach, thus introducing some of the concepts to present sound analysis studies, classification techniques and its statistical performance. Later, Section 3 summarizes different works related to past studies using acoustic classification. Then, Section 4 presents the empirical results and Section 5 concludes with remarks and discusses future work.

## 2. Material and Methods

### 2.1. Dataset

The data analyzed originates from online purchase order confirmations, made on an e-commerce website from 28 May 2018 to 28 August 2018, originating throughout Brazil. This set contains a total of 789 orders, of which 392 (49.5%) are fraud. It is important to mention that every purchase confirmation history contains information related to the buyer’s characteristics as well as a recording related to verification of purchase.

The historical data is divided into two tables, *tborder* and *tbcall*. The first shows information about the buyer (location, email domain, gender, etc.) and about the order itself, such as the date and time of the order fulfillment, price, number of installments and product category, which is quite granular, ranging from consumer electronics to furniture. Table 1 shows the features of the data set.

**Figure 1:**
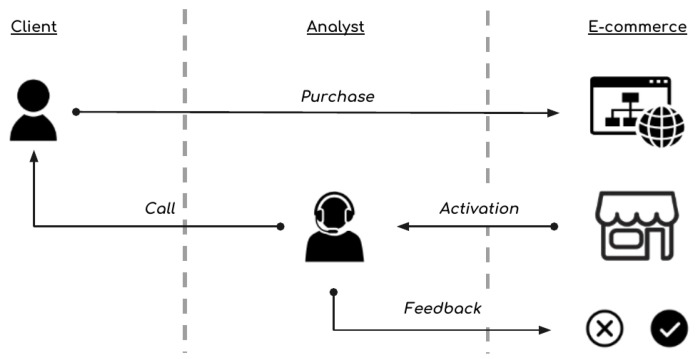
E-commerce analysis activation cycle.

The second part called *tbcall*, contains time related information regarding phone calls made by analysts that were trying to find whether the order was a fraud or not, as described in Figure 1.

Table 2 describes the phone calls and acoustic information, through the seven indexes extracted from each phone call audio file; for instance acoustic diversity index (ADI), acoustic complexity index (ACI), acoustic richness index (AR), temporal entropy (HT) index, Amplitude index (M) and other audio-related features. Greater details will be presented in the next subsection.

More information can be obtained from both tables using feature engineering process. For instance, from the “ordered on” variable the day of the week the order was placed can be obtained or even the period of the day because it has the complete date and time information. The target variable, which we would like to gain a more in-depth understanding of, is the “is fraud” variable.

### 2.2. Acoustic Indexes

Time series modeling aims to provide methods for predicting and understanding dynamic phenomena from observed data on time through modeling. Given that, real-world problems present non-deterministic models, an initial value of the process does not (often) predict the exact future value, then stochastic models are needed. Kolmogorov [4] and Whittle [5] pointed out the relationship between the prediction error variance $\left(\sigma^{2}\right)$ and the power spectrum (p(f)) of a stationary process.

Using information theoretical characterization of manner one can focus on the overall distribution of spectral power of the speech extracting audio features for modeling, see Llanos [6]. Specifically, it is hypothesized that speech production leaves a manner-specific trace in the distribution of spectral power along the decibel range that can be appropriately quantified by the Shannon entropy formula. For further details, please see Shannon [7].

Thus, an acoustic index is a statistic that summarizes aspects of a sound recording for example, the distribution of acoustic energy and other information. For this work, some acoustic indexes from many fields of study such as biodiversity were tested. Table 3 summarizes the adopted indexes.

Information obtained through audio entropy extraction (via indices used in risk management aided by classification, linear and nonlinear classifiers, responsible for associating the purchase event online to a likelihood of fraudulent purchase will be discussed in the next subsection.

### 2.3. Classification Models

The classification’ data analysis task is to obtain adaptive models or classifiers to construct or predict categorical labels, where this target variable (Y) is the label of the purchase, where 0 (zero) means if the purchase is not fraudulent and 1 (one) otherwise.

The goal of classification is to accurately predict the target class for each case in the data. The methods used for classification first predict the probability of each of the categories of a qualitative (dichotomous) variable, as the basis for making the classification [12]. Two known classifiers were chosen:

I. Logistic Regression (RL): In statistics, logistic regression is a regression model where the dependent variable has a linear dependence/relation with the predictor variables, using a function *logit*, implying this model response will always be between 0 and 1 [13].

II. Random Forest (RF): Random forests are a combination of tree predictors. Each tree depends on the values of a random vector that is sampled independently and has the same distribution for all the trees. A large number of trees is generated and they vote for the most popular class [14].

#### Performance Statistics

Since the target in this application is in the power of the prediction, the performance of the adjusted models need to be tested regardless of the used data for modelling [12]. This method is known as *cross validation*, where data points are randomly assigned to two sets called training and test set. The size of each of the sets is arbitrary although typically the test set is smaller than the training set.

After splitting the data, the models were adjusted in the training set, defining a threshold and using the obtained probability to predict the target variable label, then its performance was compared given the observed data. The model fit can be quantified through performance metrics adopted in this work, as described below.

- MCC—Matthews Correlation Coefficient. Correlation measure of binary classifications, where 1 corresponds to a perfect classifier, 0 indicates a random prediction and −1 is a completely wrong classifier. True and false positives and negatives are considered in the calculation [15].
- ROC—Receiver Operating Characteristic curve. Graphical method to evaluate, organize and select diagnostics and predictions. Based on the probability of detection (rate of true positives, axes x) against false alarms (axes y) [16].
- AUC—Area Under the ROC curve. The proportion of fraction of the area of a square of one. Indicating the positive example is ordered first as a negative example [16].
- ACCURACY—The fraction of the right predictions from the adjusted model. Rate of true and false positive over the sum of true and false positive and negative.
- SENSIBILITY—The fraction of actual positives correctly identified according to the adjusted model. Rate of true positive over the sum of true positive plus false negative.
- SPECIFICITY—The fraction of actual negatives correctly identified according to the adjusted model. Rate of true negative over the sum of true negative plus false negative.
- PRECISION—Rate of true positive over the sum of true positive and false negative.
- NVP—Negative Predictive Values. Rate of true negative over the sum of true and false negative.
- KS—Kolmogorov-Smirnov measures performance. Measure of similarity between the cumulative empirical distributions between predicted and observed data.

The classification task is common in *credit* and *behaviour score*, therefore predicting its performance is crucial to evaluate and compare the adjusted models. The next section will discuss some past works regarding the detection of fraudulent activities and vocal analysis. All the statistical analysis used the R software.

## 3. Related Works

Statistical classification methods can be applied considering the many challenging contexts of consumer credit. Over the last two decades, the number of studies in this area have increased significantly and have aimed not only at improving the models, given fraud detection presents challenging conditions such as skewed distributions and non-uniform cost per error in the data but also in techniques facing pre-processing methods [17,18,19]. Furthermore, such classification tasks have also been used in the fraud detection field for the ever-increasing e-commerce credit card scenario, see Bolton [20].

Additionally, the enrichment of the database by adopting tools describe unstructured data, such as fraudulent activity and a new area to be explored. For example, companies are increasingly generating information related to modeling linguistic and acoustic data in which they are being recorded/collected and require to be processed [21,22,23]. For instance, Burgoon [24] applied this analysis to detect fraudulent statements in company conference calls.

Burgoon et al. [24] presented a case in which linguistic analysis combined with acoustic indices were used in the description of false communication, in negotiations of public and private companies in order to investigate fraudulent actions, investors, researchers and lenders. These cases appeared more relevant in situations where the interlocutors needed to improvise the communication and this situation is more similar to the case of analyzing telephone calls for fraud detection. In this case, the vocal quality of speech is related to the situation of the speaker lying or not [24], the vibration of the vocal folds is facilitated by the muscles that surround the larynx. These muscles contract during stress and arousal states, which increases the tension that affects the frequency of vocal fold vibration.

There are many companies that analyze discourse (linguistic and vocal analysis), however it is common to realize that this work is done manually and is exhaustive and boring [25]. This problem motivated this work, as it studied the viability of entropy in these analyses in the form of acoustic indices. Although commonly applied in the biology field for example in Sueur [11] where the acoustic indexes were applied to identify the biodiversity in forests, it can also be applied in many different contexts, aiming to extract audio characteristics to help the specialists make decision (usually in the classification task).

The next section will present all three points mentioned here: *Credit/Behavior Score*, *Linguistic and Vocal Analysis* and *Acoustic Indexes* under the perspective of a study case from a company, located in São Paulo state, Brazil, which analyzes the quality of e-commerce purchases aimed to highlight possible frauds.

## 4. Results

The data analyses considered only 2018, presenting approximately a 50% fraud rate in the set of a company that operates throughout Brazil. In the first part, we conducted a descriptive analysis of the data aiming to gain some insights into the relationship between features related to the buyer’s informed characteristics.

It is worth mentioning that the database provided by the company, under study, presented the purchases as fraudulent operations or not (labeled data set). These fraudulent operations have been subject to review by an operator (assisted by the model of the company), however not classified as such and subsequently confirmed as fraudulent (once the occurrence materialized).

There are places that have a higher number of frauds reported. For example, the cities of São Paulo and Rio de Janeiro had a high rate of fraud (56.6% and 65%, respectively).

**Figure 2:**
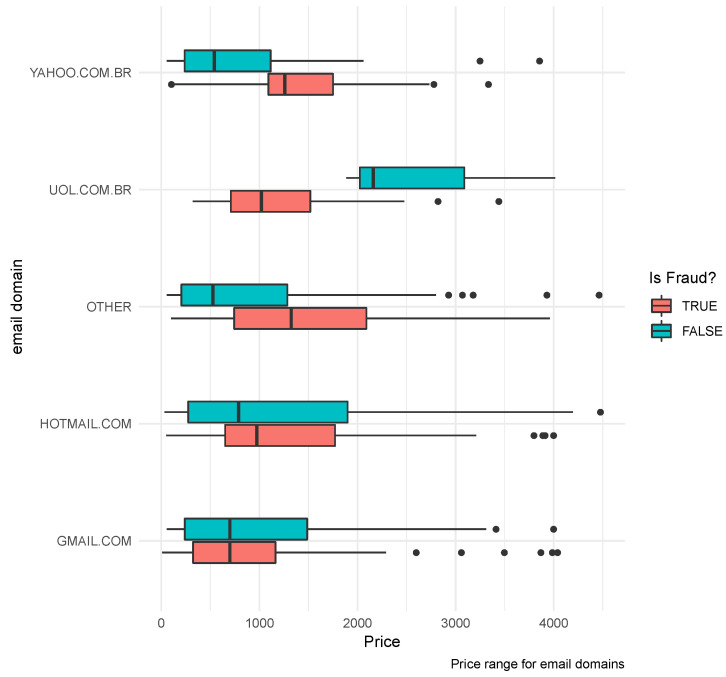
Price range boxplot per email domain. The first three domains suggest a difference pattern across the fraudulent group.

Considering the domain variable of the informed email, it helped to classify bad payers (fraudulent activities) serving as a flags-up. For instance, different quantiles for Yahoo.com.br and Uol.com.br domain were shown, according to Figure 2.

Regarding the category of the products, we found a significant difference between cell phones (which experiences the most fraud attempts) compared to the clothes category. It was also possible to identify some relations between the variables: gender and product category. Products such as cellphones, games, sporting goods and electronics were more likely to be fraud among men buyers while in the female gender, clothing products, footwear and beauty products were the most targeted. In certain categories, important facts were observed. Adding the gender dimension, for example, it was observed that in the category of mobile phones, males were associated with a higher risk even being the most recurring consumers. However, in categories such as games, when purchases were made by males, there was a much lower chance of fraud compared to when purchases were made by the females.

Noting the recurrence of fraud activity throughout the days of the week, the highest rates were observed on Thursdays (52.1%), Fridays (54.6%) and Sundays (50.9%), while a smaller difference between fraud and non-fraud activities on the other days. Thus, greater care should be taken in combining the other purchasing information in order to better classify these transactions into analyzes.

**Figure 3:**
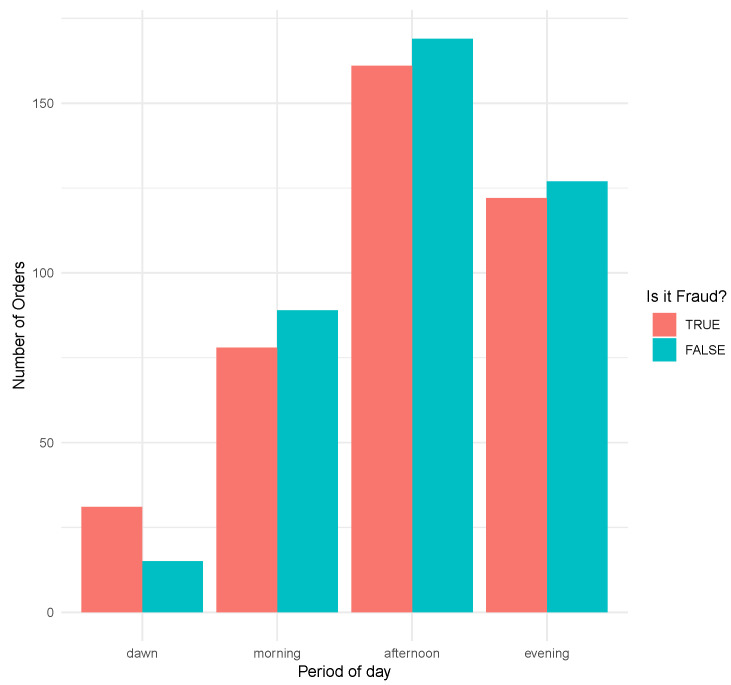
Bar plot comparing the number of orders per period. A higher fraudulent activity is suggested during the dawn.

The recurrence of frauds given the period of the day relates to the period of the order which was placed. Figure 3 discriminates some differences, especially perceiving a higher fraud incidence in orders placed at dawn. Thus, it was observed that there is an increase in fraudulent operations in the period at dawn, representing 67.4% of the activities, compared to the other times of day.

Another informative variable was the installment of the purchase made, which in practice will also be conditioned to the value of the product. It was observed that purchases with five or fewer installments have a relatively higher order value. For six or more, there is no significant difference in values.

In the second part of the case analysis, it was observed that features related to audio, for example, the duration of buyer-related audio fraudulent cases tend to be longer (average 178.12 s). In contrast, the average duration of the non-fraud audio is 118.62 s, 33.43%.

**Figure 4:**
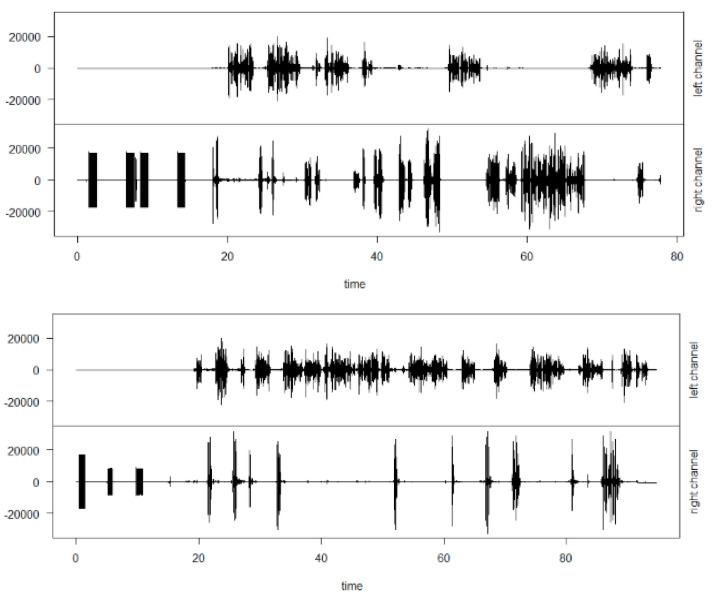
Spectrum plot from fraud (**top**) and non-fraud (**bottom**) audio channels. In both scenarios, the right channel is related to the operator voice only and the left channel related with the customer acoustic response.

Figure 4 shows the spectrogram of two audios, one related to a fraudulent activity and the other a non-fraudulent, where each audio has two channels. The left is related to the buyer’s voice and the right of the operator/company). The difference between the dynamics of the audios, observing the left channels respectively can be observed, where one is longer than the other, as well as its dynamism.

Prior to the modeling procedure, the database was divided into training (80%) and testing (20%).

Thus, using only 41 features (after transformations of some variables into dummies) two techniques were adopted; logistic regression (LR) and random forest (RF). Since a linear model was adopted, the categorical variables were transformed into dummy features, increasing to 17 “new” features. These results of processing/extraction of acoustic indices was incorporated into classification models and presented relevant results in the case study.

Four models were adjusted in this analysis; a full model using the Logistic Regression and Random Forest (using all the features) and then a reduced model for both classifiers. After concluding the full Logistic Regression and Random Forest with all the variables, we tried to reduce the number of variables through the the importance of each one on the first models.

**Figure 5:**
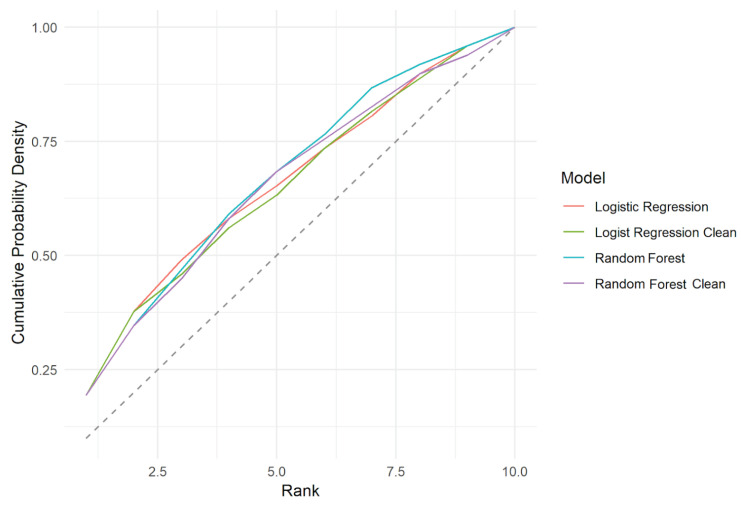
Kolmogorov–Smirnov test for classification models. Both Random Forest models presented a better performance, which is a higher area under the curve compared to the Logistic Regression models.

The adjustments of the classification models can be represented/compared by Kolmogorov- Smirnov test for classification models, as shown in Figure 5.

Considering each model optimal cut-off, some performance statistics were calculated (based on the prediction set) shown in Table 4.

**Figure 6:**
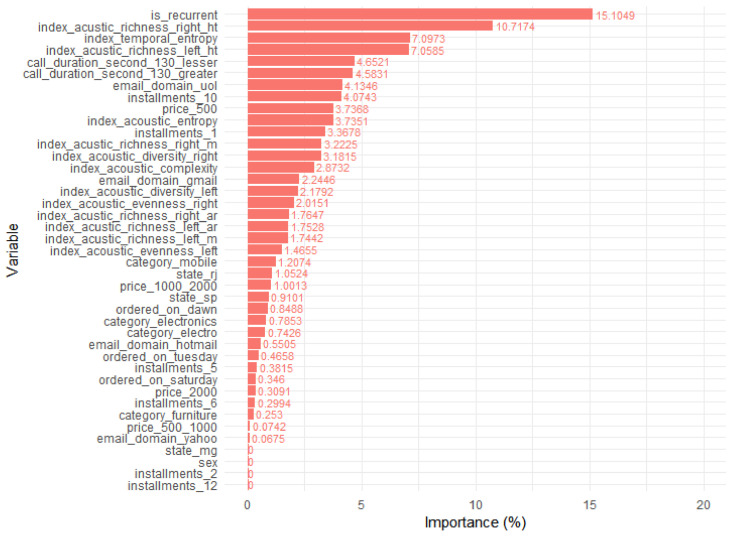
Importance of variables from the Random Forest model. Three out of the top five most important variables are related to the acoustic patterns, which are explicit through some complexity calculation transformation (entropy).

The complete logistic regression classifier indicates greater relevance to the indices acoustic; especially to acoustic richness. In fact, it can be seen see that it was the most relevant in all the classifiers tested. The random forest classifier presented a better performance, in which acoustic richness was the most important feature. Therefore, the next step was to test them again using only the indexes as the features in the model, in Figure 6. The adopted function is “varImp.RandomForest” implemented in the “caret” package. This function is related with the variables’ predictive power according to the Accuracy-based importance (prediction accuracy on the out-of-bag sample) and Gini-based importance (the node split decision of which variable). For further information, please see Reference [26].

It is important to point out that acoustic richness remained the most important feature for all the four adjusted models. After selecting the RF summary model, the company studied provided two new unlabeled audios in order to estimate the likelihood of the fraud. The final model requires information from client features (gender, email domain, frequent consumer, ordered day, ordered time, city, product, installments and price), as well as the recorded audio features extracted.

Similarly, the same models were adjusted excluding audio-related features. Comparing the models in all the scenarios (Logistic Regression & Random Forest), the metrics showed a reduction in their performance. For instance, comparing the best performing model (again Random Forest), the area under the curve (AUC) showed a reduction of −3.98%, Sensitivity (SEN) −10.1% and Specificity (SPE) −12.05%. Those finds corroborate with the results, showing that the acoustic analysis enhances the fraud detection model combined with buyer-placed information.

Finally, the last test were conducted, in which two new audios were labeled, according to the developed model, which gave a probability of being a fraudulent action (using a Random Forest classifier). The first purchase tested gave a 71.91% probability of fraud, that is, the chance of a fraudulent operation, wherein this scenario the fraud occurred. In a second purchase the model gave a 3.31% of fraud probability, where indeed it was not a fraudulent operation. Therefore, the results showed a satisfactory and promising performance and further tests shall be conducted in order to implement it in the studied company detection process.

## 5. Discussion

This study shows evidence that acoustic analysis combined with the common credit score approach shows good results towards e-commerce fraud detection. In addition, acoustic index analysis applied to speech interpretation (linguistic and vocal) explores a new area of application of entropy to problems related to industry. For example, detection of fraudulent claims or even the detection of purchases distinguishing good buyers and fraudsters, in which fraudsters hold legitimate data/ information from other people.

The acoustic indexes were, in fact, capable of providing better accuracy to the models, enhanced with the Random Forest. For the calculation of acoustic indices, we used the original audios without any pre-processing of the signals or even observed the dynamics of their complexity over time (calculate entropy of signals every 10 s for example). Thus, the results present further discussions/suggestions that may be incorporated into future work.

This work presented a limitation, addressed to the obtained results generalization, whereby to be implemented by the company (in the business intelligence tool). This manuscript finds shall be extended. We showed that models could be outperformed using acoustic analysis; nevertheless, comparison methods (cross-validation & holdout) can be incorporated into the estimation process, as a major component to enhance the models’ predictive ability. For further details, please see Reference [27]. In the meantime, the results presented remain valid given that the sample size used to obtain the results is large, ensuring good asymptotic performance in the obtained estimates [28].

The results also indicate that the developed technology could be implemented to help the human analysis of purchases still requiring further analysis in order to confirm the accuracy of the adopted model. Using this technology combined with the operators’ perception could help them make better decisions in cases of doubt if the buyer is lying or not. This study helped the company to make an analysis under two perspectives; first the model developed may reduce the number of frauds by improving the classification of fraudulent activities and, second, suggesting the adoption of data analysis unstructured as a way to enrich the database.

## Figures and Tables

**Table 1:** Features of the table tborder.

Feature	Description	Type
orderid	id of the order	integer
ordered on	date when the order was placed	date
is fraud	the target variable	boolean
is recurrent	customer has ordered before	boolean
gender	gender of the customer	char
email domain	e-mail domain that customer registered	char
state	state of the shipping address	char
city	city of the shipping address	char
neighborhood	neighborhood of the shipping address	char
category	product category	char
installments	number of installments of the order	integer
price	price of the order	real

**Table 2:** Features of the table tbcall.

Feature	Description	Type
call id	id of the call	integer
called on	date of the call	date
call duration second	duration of the call in seconds	real
is fraud	the target variable	boolean
order id	id of the order	integer
audio id	id of the audio file related to the call	integer
index acoustic complexity	acoustic complexity index	real
index acoustic diversity right	acoustic diversity index—right channel	real
index acoustic r.ar	acoustic richness index—right channel	real
index acoustic r.ht	acoustic richness index (entropy)—right channel	real
index acoustic l.ht	acoustic richness index (entropy)—left channel	real
index acoustic r.m	acoustic richness index (amplitude)—right channel	real
index acoustic l.m	acoustic richness index (amplitude)—left channel	real
index temporal entropy	temporal entropy index	real
index acoustic entropy	acoustic entropy index	real

**Table 3:** Summary of Acoustic Indexes.

Index	Affix	Summary	Reference
Acoustic Complexity Index	ACI	Average absolute amplitude difference between adjacent cells of the STDFT matrix in each frequency bin	Pieretti et al. [8]
Acoustic diversity index	ADI	Shannon entropy on the spectral content	Villanueva-Rivera et al. [9]
Acoustic richness index	AR	Ranks of the indices M and Ht obtained for a set of n files	Depraetere et al. [10]
Temporal entropy	HT	Shannon evenness of the amplitude envelope	Sueur et al. [11]
Amplitude index	M	Amplitude index that computes the median of the amplitude envelope	Depraetere et al. [10]
Acoustic Entropy	H	Multiplication of the Shannon evenness (Time vs. Frequency domain)	Sueur et al. [11]
Acoustic evenness index	G	ADI variation instead uses the Gini coefficient as measure of distribution inequality	Villanueva-Rivera et al. [9]

**Table 4:** Model statistics comparison.

Metrics	Logistic Regression	Red. Logistic Regre	Random Forest	Red. Random Forest
# Variable	41	37	41	**36**
MCC	0.2607	0.2531	**0.4808**	0.4703
AUC	0.7945	0.7978	**0.8046**	0.7983
Accuracy	0.7556	0.7575	**0.7579**	0.7525
Sensibility	0.6800	0.6835	0.6951	**0.7123**
Specificity	0.8281	**0.8288**	0.8200	0.7889
Precision	0.8058	**0.8069**	0.8042	0.7805
NVP	0.7229	0.7251	0.7277	**0.7379**
KS	0.4882	0.4906	**0.5000**	0.4858
